# iTRAQ protein profile analysis of developmental dynamics in soybean [*Glycine max* (L.) Merr.] leaves

**DOI:** 10.1371/journal.pone.0181910

**Published:** 2017-09-27

**Authors:** Jun Qin, Jianan Zhang, Fengmin Wang, Jinghua Wang, Zhi Zheng, Changcheng Yin, Hao Chen, Ainong Shi, Bo Zhang, Pengyin Chen, Mengchen Zhang

**Affiliations:** 1 National Soybean Improvement Center Shijiazhuang Sub-Center. North China Key Laboratory of Biology and Genetic Improvement of Soybean Ministry of Agriculture, Cereal & Oil Crop Institute, Hebei Academy of Agricultural and Forestry Sciences, Shijiazhuang, P.R. China; 2 Department of Crop, Soil and Environmental Sciences, University of Arkansas, Fayetteville, AR, United States of America; 3 National Foxtail Millet Improvement Center, Minor Cereal Crops Laboratory of Hebei Province Institute of Millet Crops, Hebei Academy of Agriculture and Forestry Sciences, Shijiazhuang, P. R. China; 4 Beijing Protein Innovation, B-8, Beijing Airport Industrial Zone, Beijing, People’s Republic of China; 5 Department of Crop, Soil and Environmental Sciences, Virginia Tech, Blacksburg, VA, United States of America; Chinese University of Hong Kong, HONG KONG

## Abstract

Zao5241 is an elite soybean [Glycine max (L.) Merr.] line and backbone parent. In this study, we employed iTRAQ to analyze the proteomes and protein expression profiles of Zao5241 during leaf development. We identified 1,245 proteins in all experiments, of which only 45 had been previously annotated. Among overlapping proteins between three biological replicates, 598 proteins with 2 unique peptides identified were reliably quantified. The protein datasets were classified into 36 GO functional terms, and the photosynthesis term was most significantly enriched. A total of 113 proteins were defined as being differentially expressed during leaf development; 41 proteins were found to be differently expressed between two and four week old leaves, and 84 proteins were found to be differently expressed between two and six week old leaves, respectively. Cluster analysis of the data revealed dynamic proteomes. Proteins annotated as electron carrier activity were greatly enriched in the peak expression profiles, and photosynthesis proteins were negatively modulated along the whole time course. This dataset will serve as the foundation for a systems biology approach to understanding photosynthetic development.

## Introduction

Soybean [Glycine max (L.) Merr.] is an important oil and grain crop and is also the world's most important source of edible vegetable oil and vegetable protein[1]. Soybean seeds are rich in protein, fat, and other nutritional compounds including isoflavones and oligosaccharides[2]. With the increasing gulf between supply and demand, fully tapping yield potential and producing elite, high yielding, high quality soybean cultivars is essential for production[3]. Photosynthetic efficiency directly affects soybean production, so understanding the dynamic development of the leaf is of singular importance.

As the leaf is so important to the basic functions of plant growth including photosynthesis, transpiration, and respiration, it has been the focus of widespread and continuing research. The cell-specific transcriptomes of successive developmental stages were compared in bundle sheath (BS) and mesophyll (M) cells of maize leaves [4], the results showed that the number of genes preferentially transcribed in one or another cell type varies greatly between the different stages of leaf development. The transcriptome of maize leaves were analyzed using Illumina sequencing, the results quantified transcript abundance along a leaf developmental gradient in mature BS and M cells [5]. Differential gene expression analysis was performed in soybean leaf tissues at late developmental stages under drought stress showing that the down regulation of many photosynthesis-related genes can contribute to retardation of growth under drought stress which may serve as an adaptive mechanism for plant survival[6]. However, the research relevant to the dynamic development of soybean leaves is still lacking.

Studying proteins to reveal gene function and the nature of biological phenomena is important. However, biological proteins are complex, and each cell generally has thousands of proteins. Therefore, a technique capable of separating large number of proteins simultaneously is required. Proteomics is a recent developing technology which could be useful for the large-scale, comprehensive study of protein structure, modification, action and expression in cells and tissue [7]. Comparative proteomics is the identification of differences and changes in the proteome between states, tissue types, and environments. On one level, this provides the tools and methods for studying the function of life and physiological and pathological phenomena, but these same tools can also be applied to examine the basic laws of life [8–10]. There are many methods for proteomics research. Two-dimensional electrophoresis (2-DE) is the main method for separating proteins due to its simplicity and is widely used in various plants proteomics. However, 2-DE results are not sufficiently accurate with some proteins being easily lost and others hard to detect in low abundance [11]. Compared with 2-DE, isotope affinity tag (ICAT) technology significantly improves the ease of separating membrane proteins, can be combined with high performance liquid chromatography, and provides protein isolation results which are more accurate and reliable. However, ICAT technology applies only to proteins containing cysteine residues and identifying small molecule peptides is difficult [12]. iTRAQ (Isobaric Tags for Relative and Absolute Quantitation) is a high-throughput method which can be used to study the relative and absolute quantification of 2–8 samples at the same time with good accuracy and repeatability. iTRAQ is one of the most widely used markers in comparison proteomics [13]. The research technology of iTRAQ quantitative protein has high sensitivity and can identify any type of protein. iTRAQ does not affect protein physical and chemical properties such as the isoelectric point, abundance of the protein itself, molecular weight, and hydrophobicity. At the same time, iTRAQ can detect alkaline, hydrophobic, high or low molecular weight, and low abundance proteins which are difficult to detect using 2-DE. In protein quantitative experiments using iTRAQ, more than 97% of the peptides can be marked efficiently and completely with high repeatability. In addition, through direct tandem application with the mass spectrum, iTRAQ can be automated saving time and effort with a fast reaction rate. All in all, iTRAQ is a good technique to study the plant protein quantity.

In our previously study, we compared leaf proteome specifically the photosynthetic protein expression patterns, between Hobbit and JD17 at same developmental stage [14]. In this research, we compared the protein different expression profiles in three important growth periods of Zao 5241, like V2 (second Trifolicate), R1 (beginning flowering) and R3 (beginning pod). Moreover, we analyzed enriched expressed proteins and revealed dynamic proteomes by cluster analysis. The results reported here represent a necessary step toward providing a useful tool and information for isolating proteins and better understanding the molecular basis underlying developmental dynamics in soybean.

## Materials and methods

### Plant growth conditions and tissue collection

Zao 5241 is one of the most important elite germplasm lines for soybean breeding in the Huang-Huai-Hai region incorporating foreign descent such as Yanli from Japan and Williams from the United States. It is a versatile line with excellent plant type traits, early maturity, high oil content (23.77%), good resistance to four different strains of Soybean Mosaic Virus (SMV), and combining ability. It has been used to develop new soybean varieties and germplasm for twenty years in summer soybean breeding.

The plants were grown in a 50:50 mix of vermiculite and soil in a greenhouse with 12hr/12hr light/dark, 30°C light/ 22°C dark, and 60% relative humidity in Shijiazhuang (114°26’E, 38°03’N). Three growth stages, V2 (second trifolicate), R1 (beginning flowering) and R3 (beginning pod) were chosen as time points to analyze the protein expression profiles in order to analyze and detect different protein expression profiles associated with leave, flower and pod development. Leaves were collected at 2, 4, and 6-weeks 2 hours into the light period. Three biological replicates were carried out, and each sample was pooled from 5 plants.

### Protein extraction

Total protein samples were extracted using the cold-acetone method described by Meyer et al. [15]. Leaves were ground in liquid nitrogen and suspended in precooled acetone (-20°C) containing 10% (v/v) TCA. Then, samples were incubated at -20°C for 2 h after thorough mixing. Proteins were collected by centrifuging at 30,000 g and 4°C for 30 min, and the supernatant was carefully removed. To reduce acidity, the protein pellets were re-suspended with precooled acetone and centrifuged again at 20,000 g and 4°C for 30 min. They were then washed 3 times with cold acetone and re-suspended in 1 ml protein extraction reagent [8 M urea, 4% (w/v) CHAPS, 30 mM HEPES, 1 mM PMSF, 2 mM EDTA and 10 mM DTT], the protein was resolubilized through sonication in an ultrasonic cleaner (SCIENTZ, SB4200D). The supernatant was collected after centrifuging at 20,000 g at 4°C for 30 min. Protein concentration was determined using a 2-D Quant Kit (General Electric Company, USA). SDS-PAGE was used to verify the protein quality and concentration.

### Protein digestion and iTRAQ labeling

Total protein (100 μL) was diluted by equal volume tetraethylammonium bicarbonate (TEAB, pH 8.5; Sigma, St. Louis, MO). Subsequently, modified trypsin (Promega, Madison, WI) was added to the mixture (3.3 μg trypsin/100 μg protein) to digest the protein at 37°C for 24 hour. The solvent was then removed in a refrigerated centrifuge (Eppendorf, 5417R), and MALDI TOF/TOF (Bruker limited, Coventry, UK) was used to verify the efficiency of protein digestion.

Peptides were labeled with an iTRAQ labeling kit (Applied Biosystems) according to the manufacturer’s manual. 2, 4, and 6-week-old samples were labeled with reagent 114–119, respectively. Nine Peptide samples labeled were divided into two iTRAQ groups, each iTRAQ groups were carried out independently (S1 Table). Three independent biological experiments were performed. The peptides were then combined and further fractionated offline using strong cation exchange (SCX) with high performance liquid chromatography (HLPC) system (Shimadzu, Japan) connected to a SCX column (Luna 5 μ column, 4.6 mm I.D. × 250 mm, 5 μm, 100 Å; Phenomenex, Torrence, CA). The retained peptides were eluted using Buffer A (10 mM KH_2_PO_4_ in 25% ACN, pH 3.0) and Buffer B (2 M KCl, 10 mM KH_2_PO_4_ in 25% ACN, pH 3.0) with a flow rate of 1 mL/min. In total, 38 fractions were collected and combined into 17 fractions to reduce peptide complexity. Subsequently, eluted fractions were lyophilized in a centrifugal speed vacuum concentrator and dissolved with 0.1% formic acid prior to reverse-phase nanoflow liquid chromatography (nLC) and tandem mass spectrometry (nLC-MS/MS).

### MS/MS analysis

MS/MS analysis was performed on an Easy Nano-LC system (Proxeon Biosystems, Odense, Denmark) connected to a hybrid qradrupole/time-of-flight mass spectrometer (MircOTOF-Q II, Bruker, Germany) equipped with a nano electrospray ion source. Peptides of each fraction were equalized before being injected into the Nano-LC-system. The peptides were separated on a C18 analytical reverse phase column of 75μm i.d.x100mm length with Solution A (5% acetonitrile, v/v, 0.1% formic acid, v/v), and Solution B (95% acetonitrile, v/v, 0.1% formic acid, v/v) at a flow rate of 300 nL/min. After equilibrating with 5% Solution B for 10 min, the following gradient schedule would start: 45% Solution B at 80 min, 80% Solution B at 85 min, maintained for 15 min, 5% at 105 min and held for 15 min before ramping back down to the initial solvent condition. The MicroTOF-Q II hybrid MS was used to analyze the fractions. The MS/MS survey scan for all experiments between 50 and 2000 mass-to-charge ratio (m/z). The ionization tip voltage and interface temperature was run as 1250 V and 150°C.

### Protein identification and quantification

All MS data were collected using MicrOTOFcontrol and analyzed using DataAnalysis (Bruker Daltonics). Mascot v2.3.01 (Matrix Science) [16] was used to identify proteins. The Universal Protein Resource (UniProt) [17] database was used as a reference. For biological repeats, spectra from 17 fractions were combined into one file and searched. The parameters were set as follows: specifying trypsin as the digestion enzyme; cysteine carbamidomethylation as fixed modification; iTRAQ8-Plex on N-terminal residue, iTRAQ-8Plex on tyrosine (Y), iTRAQ-8Plex on lysine (K), glutamine-pyroglutamic acid and oxidation on methionine (M) as the variable modification; peptide tolerance was set at 0.05 Da, and MS/MS tolerance was set at 0.05Da. Finally, an additional filter before exporting the data was set: significance threshold *P*<0.05 (with 95% confidence) and ion score or expected cut-off less than 0.05 (with 95% confidence).

The protein search results were exported by Mascot then normalized and quantified using Scaffold v3.0 [18]. 4- and 6-week-old protein profiles were quantified based on the 2-week-old results.

### Protein annotation

Based on the results of the protein search against the UniProt database, DAVID Bioinformatics Resources v6.7 [19] was employed to obtain the functional classification of the proteins based on Gene Ontology (GO) [20] and Clusters of Orthologous Groups (COG) terms [21] and determine the distribution of the gene functions at the macro level. The Kyoto Encyclopedia of Genes and Genomes (KEGG) database (http://www.genome.jp/kegg/) [22, 23] was used to annotate the pathway of these proteins. The enrichment test of functional category proteins was performed by a chi-square test with the defining cutoff as 0.01 under an *Arabidopsis thaliana* background. A false discovery rate (FDR) significance threshold of 0.05 was used as false-positive control.

### Differentially expressed protein definition and cluster analysis

Proteins with significant changes during leaf development were selected using the method described before [24, 25]. 90% confidence (Z-score = 1.645) of log^2^ ratios was used to select the proteins whose distribution was removed from the main distribution. The mean and SD from those proteins overlapping in three biological repeats was calculated. The profiles from different time points were calculated independently, and a broad threshold was selected. The result showed the cut-off value for up-regulated proteins was 1.75-fold (mean + Z-score × SD) and down-regulated proteins was 0.69-fold (mean—Z-score × SD). Protein ratios outside this range were defined as being significantly different at *P* = 0.01.

The expression profiles of the differentially expressed proteins were determined by cluster analysis based on the k-means method with Pearson’s correlation distance in Genesis v1.7.6 [26, 27]. The number of clusters was determined using the Figures of Merit (FOM) algorithm [28].

## Results

### Identification of leaves proteins

A total of 1,245 proteins were identified in all experiments with the number of unique peptides ranging from 0 to 40. Of all proteins, only 405 (32.53%) had been well annotated previously under a *Glycine max* background. These were mostly composed of metabolic pathway, biosynthesis of secondary metabolites pathway, and cytoplasm component proteins. Remarkably, a total of 49 *Glycine* annotated proteins related to photosynthesis were found among the abundant proteins. All of the proteins were also compared against the UniprotKB/Swiss-Prot *Arabidopsis thaliana* protein database using blastx with an E-value cut-off of 1e-5 revealing 1,212 proteins. For the three biological replicates, 1,002, 982, and 970 proteins (80.48% 78.88%, and 77.91% of total proteins) were identified with an overlap of 727 (72.55%, 74.03%, and 74.95%) proteins. Although the lack of protein information limited identification, this investigation demonstrated the capability and applicability of the iTRAQ technology to quantify the leaf proteome of soybean.

### Functional classification and enrichment analysis of proteins

**Fig 1:**
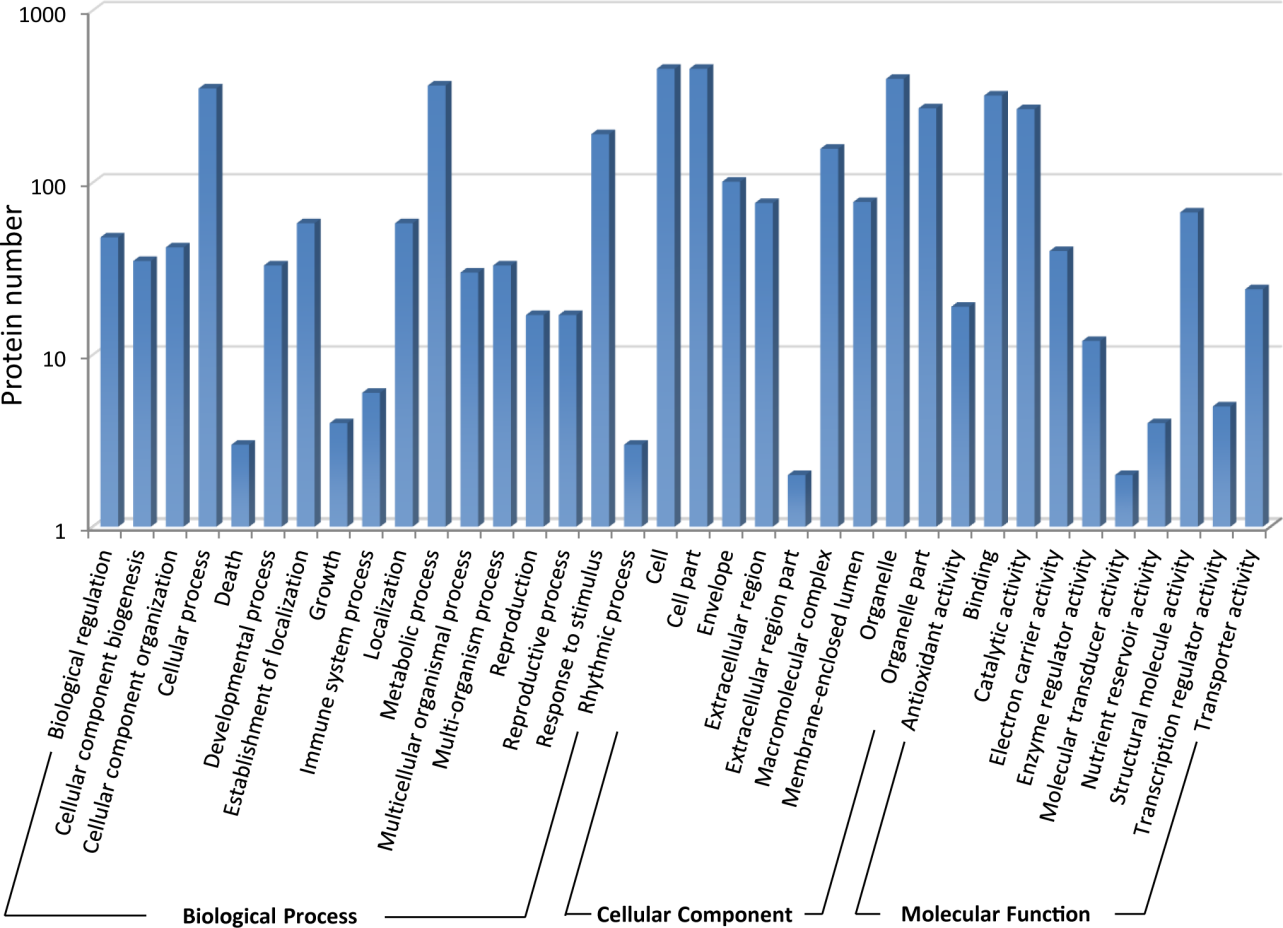
Functional classification of the iTRAQ identified proteins of soybean leaves.

For the iTRAQ dataset, 598 proteins, with at least 2 unique peptides, were used for further analysis. The iTRAQ identified proteins were classified into 36 GO functional terms which are distributed into three main categories according to their functional role: biological process (434), cellular components (460), and molecular function (439) (Fig 1).

There were 17 GO terms in the biological process category. Four out of those GO terms were significantly enriched (*P*<0.01): metabolic process (366, *P* = 4.61E-20), response to stimulus (191, *P* = 1.99E-17), cellular process (352, *P* = 1.78E-13), and cellular component biogenesis (35, *P* = 2.11E-04). Within the cellular component category, there were 9 GO terms, six of which were significantly enriched (*P*<0.01): organelle part (270, *P* = 4.87E-100), organelle (400, *P* = 1.68E-54), envelope (101, *P* = 2.05E-41), macromolecular complex (157, *P* = 1.16E-33), membrane-enclosed lumen (77, *P* = 9.09E-27), and extracellular region (76, *P* = 3.71E-11). Four out of the ten GO Terms were enriched in the molecular functional category: structural molecule activity (67, *P* = 2.17E-26), antioxidant activity (19, *P* = 3.22E-08), electron carrier activity (40, *P* = 1.56E-06), and catalytic activity (267, *P* = 1.59E-0.5). A total of 58 proteins out of 598 were annotated with photosynthesis (GO: 0015979), a child term of the biological process category, which was significantly enriched in this dataset (*P* = 1.70E-44). 31 proteins were classified as electron transport chain (GO: 0022900) which is the father category of photosynthetic electron transport chain (GO: 0009767) and respiratory electron transport chain (GO: 0022904).

Based on the KEGG database, 394 proteins were annotated with 85 pathways. Among these pathways, metabolism (gmx01100) and biosynthesis of secondary metabolites (gmx01110) involved the most proteins (163 and 85 proteins, respectively); however, photosynthesis (gmx00195) was the most enriched pathway (*P* = 2.68E-15) followed by carbon fixation in photosynthetic organisms (gmx00710) (*P* = 8.70E-10) and photosynthesis—antenna proteins (gmx00195) (*P* = 1.04E-5). In addition, another pathway related to photosynthesis, citrate cycle (TCA cycle; gmx00020), was also enriched (*P* = 1.73E-3) in the iTRAQ protein dataset (S2 Table).

### Differentially expressed proteins and enrichment analysis

**Fig 2:**
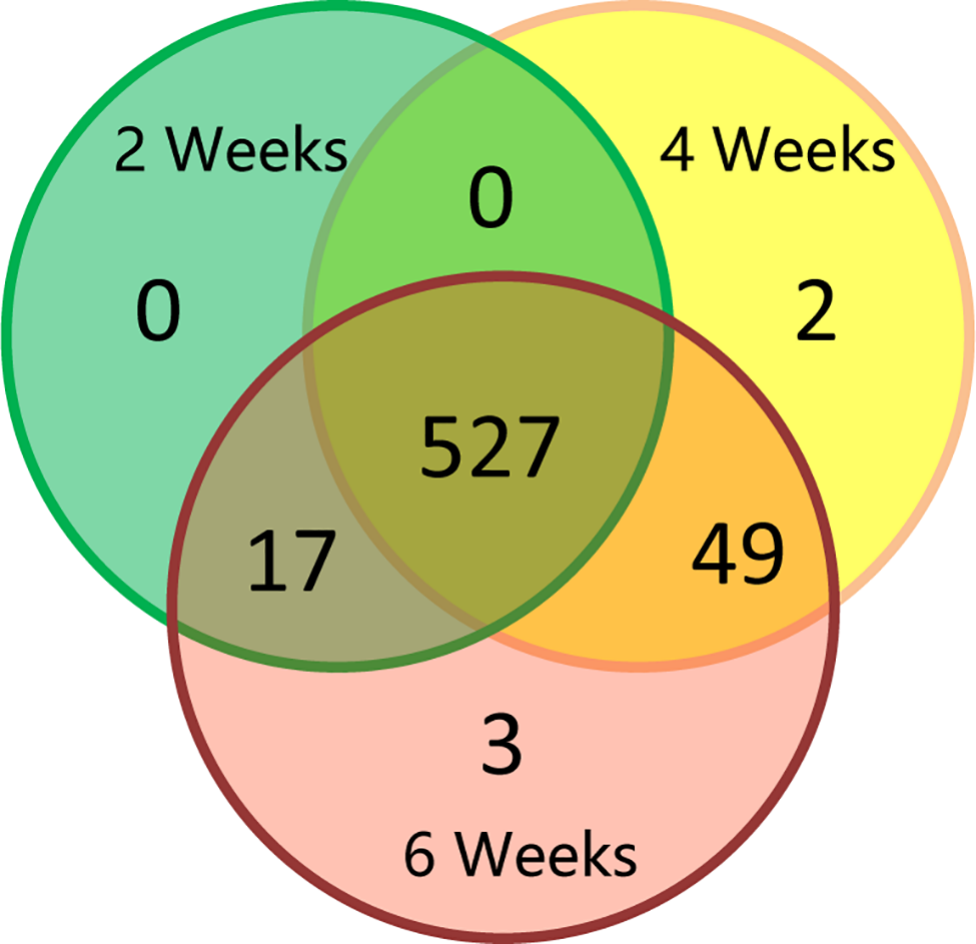
Venn diagram showing the proteins expressed in three stages of soybean development.

For 598 proteins, at least 2 unique peptides were identified which permitted quantification [24]. Within the dataset, 544, 578, and 596 proteins were identified in the two, four, and six-week stages, respectively (Fig 2).

**Fig 3:**
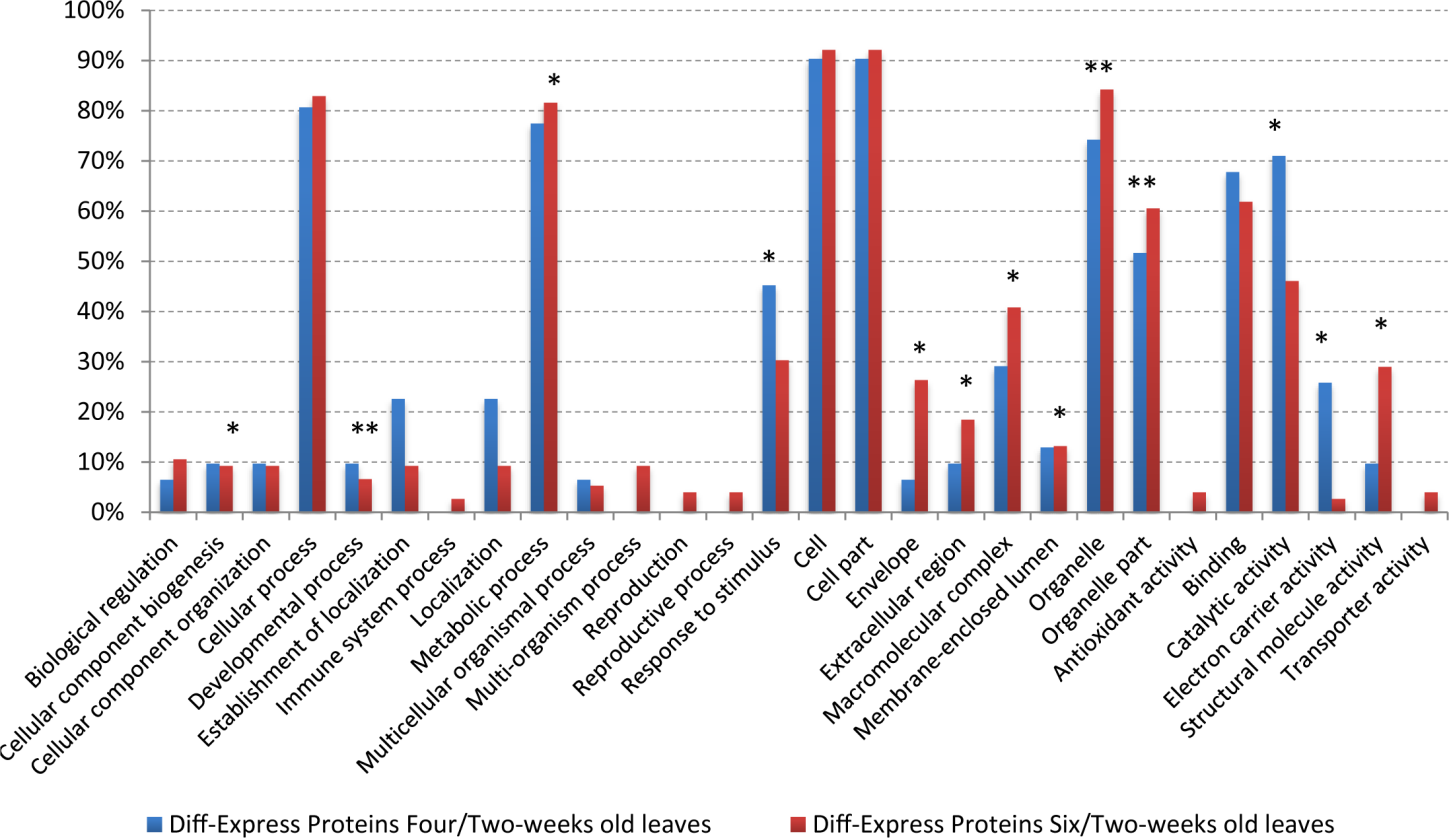
The functional classification of differentially expressed proteins in four/two and six/two weeks old leaves. * The diff-expressed proteins were enriched in this functional category.

Based on a 90% confidence level, cutoff values of 1.75-fold for up-regulated proteins and 0.69-fold for down-regulated proteins were used to define the differential expression during soybean development. A total of 113 proteins were defined as differentially expressed proteins (S3 Table). 41 proteins were found to be differentially expressed between four-week and two-week old leaves; of these, 21 were decreased and 20 increased during leaf development. 84 proteins were defined as being differentially expressed between six- and two-week old leaves; 60 of which were decreased and 24 increased during leaf development. The proteins which were differentially expressed at either four-/two- or six-/two- week old leaves were separated into categories (Fig 3). Two-week-old results serve as a reference, developmental process (GO:0032502), Organelle (GO:0043226), and organelle part (GO:0044422) protein were both enriched in four-(P = 8.39E-01, P = 2.35E-03, and P = 2.57E-06) and six-week old leaves(P = 9.70E-01, P = 4.23E-11, and P = 7.44E-20). Response to stimulus (GO: 0050896), catalytic activity (GO: 0003824) and electron carrier activity (GO: 0009055) proteins were individually enriched (P = 9.35E-3, P = 7.99E-3, and P = 7.83E-5) in four -week old leaves. Cellular component biogenesis (GO:0044085), metabolic process (GO:0008152), envelope (GO:0031975), extracellular region (GO:0005576), macromolecular complex (GO:0032991), membrane-enclosed lume (GO:0031974), and structural molecule activity (GO:0005198) were individually enriched (*P* = 6.61E-02, *P* = 2.50E-05, *P* = 1.52E-10, *P* = 1.57E-03, *P* = 2.75E-10, *P* = 1.82E-03, and *P* = 1.00E-15) in six -week old leaves.

For KEGG analysis, those proteins were significantly enriched in Photosynthesis pathway (P = 4.18E-4 for diff-expressed proteins of four/two weeks old leaves, P = 5.45E-6 for diff-expressed proteins of six/two weeks old leaves).

### Cluster analysis of differentially expressed proteins and validation with immunoassays

**Fig 4:**
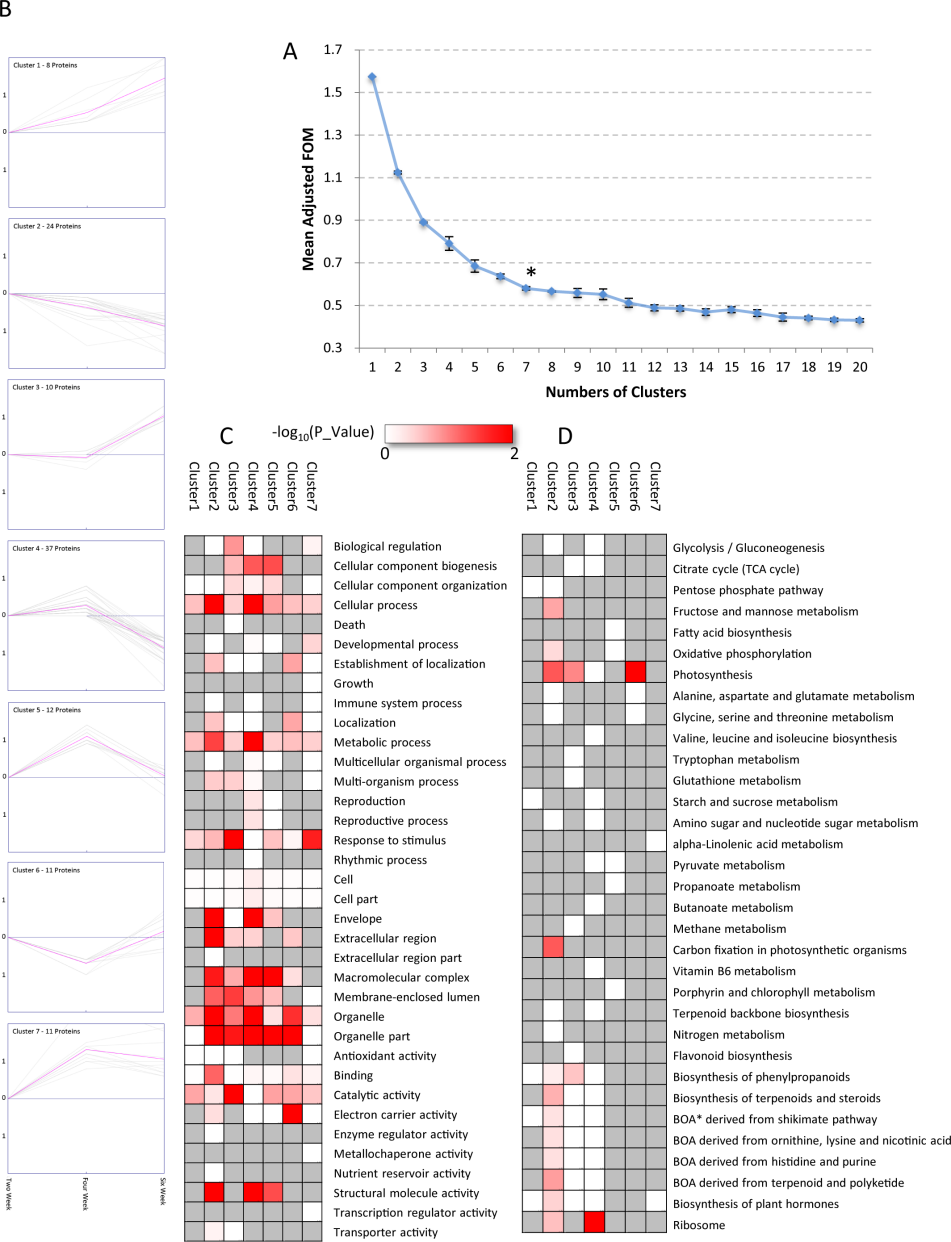
Categories distribution in the seven expression clusters. A) Figure of merit analysis indicated the number of cluster; B) Clusters were obtained by *K*-means method on the gene expression profiles from 113 diff-expressed proteins. C) Enrichment of KEGG analysis among the seven clusters. * BOA, Biosynthesis of Alkaloids. Red, significant enrichment; white, non-significant; gray, not-detected.

Clusters of differentially expressed proteins were analyzed based on the k-means methods using Pearson’s correlation distance in Genesis v1.76. The proteins were divided into seven clusters based on their expression modulation representing the number of profiles indicated by FOM analysis (Fig 4A). Cluster 1 and 2 contained proteins positively or negatively modulated along the whole time course; cluster 3 and cluster 4 contained proteins modulated only during the four to six weeks developmental stage; cluster 5 and cluster 6 contained proteins positively and negatively modulated only at four weeks old leaves, and proteins up-regulated during two to four weeks stage fell into cluster 7 (Fig 4B). Functional categories enrichment were showed in Fig 4C. For example, proteins classified into catalytic activity were greatly enriched in cluster 3, with the proteins being upregulated during the second stage. Differentially-expressed proteins annotated as electron carrier activity were greatly enriched in cluster 6 with peak expression of proteins in the four week old leaves. For each cluster, the enrichment of KEGG analysis based on the DAVID database (Fig 4C) were showed Photosynthesis pathway was significantly enriched in cluster 2 and cluster 5.

**Fig 5:**
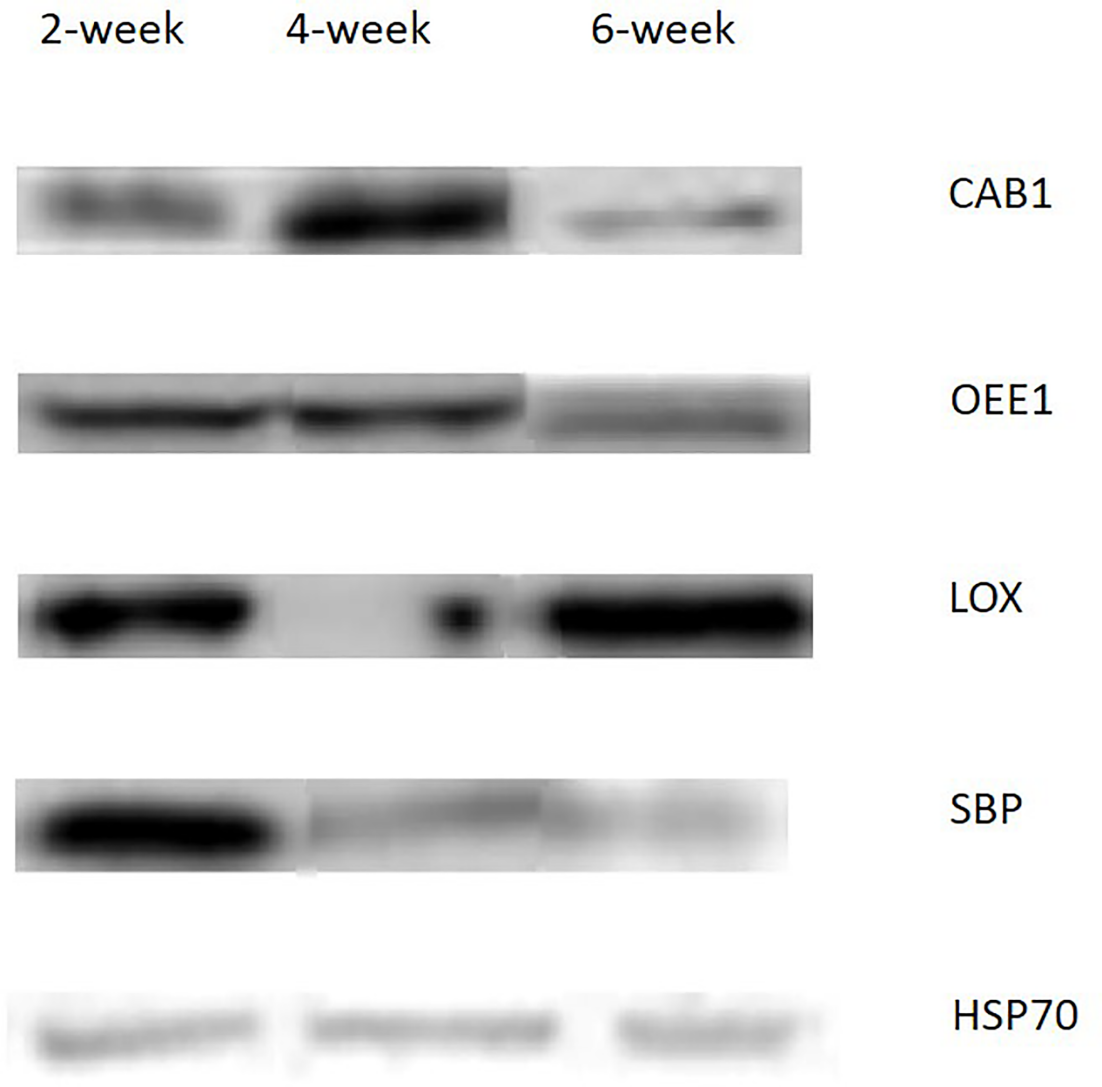
Western blotting detection of soybean proteins CAB1 OEE1 LOX SBP HSP70.

For further validation, the expression patterns of four proteins, CAB1, OEE1, LOX, and SBP were identified by western blotting and the results were consistent with the iTRAQ results, this verified the reliability of the iTRAQ system and the data analysis methods (Fig 5).

## Discussion

iTRAQ is a technique with high sensitivity and accuracy, demonstrating a remarkable advantage in simultaneous analysis of multiple samples and subsequently providing the relative quantification on hundreds of proteins at one time[29]. In the present study, an iTRAQ-based proteomic technique was used to assess proteomic changes and identify proteins which were differentially expressed during soybean leaf development. In total, 1,245 proteins were identified. Data from three biological replicates were analyzed and proteins detected by querying data with a soybean protein database. Although soybean protein annotation is limited, a total of 58 proteins out of 598 were annotated with photosynthesis, and 31 proteins were classified as electron transport chain. Pathway analysis showed that photosynthesis was the most enriched pathway followed by carbon fixation in photosynthetic organisms and photosynthesis. Differential proteomic analysis of grapevine leaves revealed responses to heat stress and subsequent recovery by iTRAQ. One hundred and seventy-four proteins were differentially expressed under heat stress and/or during the recovery phase, in comparison to unstressed controls. Based on MapMan ontology, functional categories for these dysregulated proteins included mainly photosynthesis (about 20%), proteins (13%), and stress (8%). some proteins related to electron transport chain of photosynthesis, antioxidant enzymes, HSPs and other stress response proteins, and glycolysis may play key roles in enhancing grapevine adaptation to and recovery capacity from heat stress. In our preliminary research, proteomic analysis of two soybean cultivars were performed using iTRAQ-base quantitative. Enrichment factor analysis indicated that proteins involved in photosynthesis comprised an important category[14].

To estimate how many and what proteins expressed during the leave developing, the 2-week Protein Profile of Zao5241 was used to produce the reference, 41 and 84 of 1,245 differentially expressed proteins were found in 4 weeks and 6 weeks, respectively. On the basis of GO and pathway enrichment analysis, we concluded that the “photosynthesis was most significantly enriched. KEGG and enrichment analysis of differentially expressed proteins showed that electron carrier activity proteins were significantly enriched in the photosynthesis pathway (P = 4.18E-4 for differentially expressed proteins of four-/two- weeks old leaves, P = 5.45E-6 for diff-expressed proteins of six/two weeks old leaves). The proteomes of soybean leaves and roots under salt treatment were investigated using iTRAQ-based proteomic approach. Protein-protein interaction analysis revealed metabolism, carbohydrate and energy metabolism, protein synthesis and redox homeostasis could be assigned to four high salt stress response networks[30].

Moreover, dynamic proteomes were revealed by cluster analysis. Electron carrier activity proteins were greatly enriched in the peak expression profiles, and photosynthesis proteins were negatively modulated along the whole time course. Further studies for protein function analysis were needed to further clarify the molecular mechanism during leaf development in soybean.

## Supporting information

S1 Table The information of nine peptide samples labeled. (XLSX)

S2 Table Proteins annotated with three pathways related to photosynthesis. (XLSX)

S3 Table Differentially expressed proteins between four-week and two-week old leaves. (XLSX)
